# Evaluation of electrophysiological biomarkers in the Amazonian fish *Hyphessobrycon heterorhabdus* after menthol exposure

**DOI:** 10.1007/s00360-026-01694-9

**Published:** 2026-07-20

**Authors:** Yure Jefferson da Cruz do Nascimento, Danilo Serrão Moutinho, Sildiane Cantanhêde-Magno, Moisés Hamoy, Lílian Lund Amado

**Affiliations:** 1 Laboratório de Ecotoxicologia and Laboratório de Pesquisa em Monitoramento Ambiental Marinho, Universidade Federal do Pará, Belém, PA Brazil; 2 Programa de Pós-Graduação em Farmacologia e Bioquímica, Instituto de Ciências Biológicas, Universidade Federal do Pará, Belém, PA Brazil; 3 Programa de Pós-Graduação em Ecologia, Instituto de Ciências Biológicas, Universidade Federal do Pará, Belém, PA Brazil; 4 Laboratório de Farmacologia e Toxicologia de Produtos Naturais, Instituto de Ciências Biológicas, Universidade Federal do Pará, Belém, PA Brazil

**Keywords:** Ecotoxicology, Amazon, Natural anesthetics, Physiological parameters, Sublethal effects

## Abstract

Electrophysiology is an emerging approach in the context of environmental toxicology, where it represents a non-invasive biomarker that has been little studied in tropical fish. In this study, we evaluated electrocardiographic (ECG) and electromyographic (EMG) responses in *Hyphessobrycon heterorhabdus*, a native Amazonian species considered a promising biological model for ecotoxicological studies, and Menthol was selected as the pharmacological model for methodological validation in this species. Fish were exposed to 30 mg L^− 1^ for three minutes and subsequently transferred to clean water to assess recovery. The ECG showed a reduction in heart rate and an increase in the wave intervals in fish exposed to menthol. The EMG recorded the occurrence of transient muscle spasms before a progressive decrease in contraction strength as anesthesia deepened. After recovery, both ECG and EMG tracings returned to baseline conditions, indicating the reversibility of menthol-induced anesthesia. Thus, electrophysiological markers in *H. heterorhabdus* proved to be effective tools for detecting short-term physiological changes and supports their application in studies involving environmental stressors or chemical exposures.

## Introduction

The Amazon region is subjected to multiple anthropogenic pressures, including agriculture (Dos Santos et al. 2018) and mining activities (Donoghue et al. 2014). These activities contribute to the discharge of diverse chemical compounds into aquatic ecosystems via runoff (Mayes et al. 2016). These substances can affect aquatic organisms, including fish, at several biological levels, leading to biochemical, physiological, and behavioral changes. These responses can serve as biomarkers and help in the early detection of environmental impacts (Hampel et al. 2016).

Electrophysiology has emerged as a promising approach for evaluating xenobiotic toxicity in fish, providing near-immediate detection of physiological disturbances (Cantanhêde et al. 2020). Electromyographic (EMG) analysis enables the assessment of muscle contraction and relaxation levels and frequencies (De Souza et al. 2025), while electrocardiographic (ECG) measurements quantify cardiac electrical activity by analyzing its waveform components (P, Q, R, S, and T) and cardiac intervals (RR, QRS, PQ, and QT), thereby revealing changes in cardiac function and contractile efficiency (Cantanhêde et al. 2020). Behavioral assessments complement electrophysiological analysis and represent commonly employed methodologies for validating the effects of chemical agents in experimental animals (De Araújo et al. 2021).

The use of substances with well-characterized physiological effects is crucial for validating the effectiveness of new techniques and refining analytical protocols. Anesthetics are suitable candidates for this purpose because they modulate central nervous system activity, and induce changes in both electrophysiological and behavioral patterns in fish (Vilhena et al. 2019; Cerqueira et al. 2025). As example, Tricaine methanesulfonate (MS-222) is widely recognized as the standard anesthetic for fish (Reis et al. 2025). However, alternative anesthetics have been explored for specific applications, including menthol, a low-cost, naturally derived compound extracted from the essential oil of plants of the genus *Mentha* spp., with antiseptic and anti-inflammatory properties (Lawrence 2006; Gonçalves et al. 2008). Here, menthol is employed exclusively as a pharmacological tool for methodological validation, due to its well-documented effects on fish physiology and behavior. Previous studies reported menthol effects in different endpoints, such as the growth performance in Nile Tilapia (*Oreochromis niloticus*) (Magouz et al. 2021), oxidative stress in zebrafish (*Danio rerio*) embryogenesis (Carneiro et al. 2022). In addition, the changes in cardiac responses of the amazonian fish Tambaqui (*Colossoma macropomum*) during short-term anesthesia (Cantahêde et al. 2022) were also evaluated through electrophysiological markers, however, the use of menthol was focused on fish farming management.

Several studies have evaluated the anesthetic effect of natural substances extracted from plants on Amazonian fish by assessing electrophysiological biomarkers. In *C. macropomum*, the efficacy of the anesthetic effect has already been verified through electrophysiological studies of substances such as Spilanthol (Garcia et al. 2024), essential oil of *Curcuma longa* (Cerqueira et al. 2025), and *Nepeta cataria* (Da Paz et al. 2024). Furthermore, Cantanhêde et al. (2020) evaluated clove oil-induced anesthesia in the fish *Bryconops caudomaculatus* using ECG patterns and suggested the application of this tool in ecotoxicological approaches for the assessment of contaminants.

*Hyphessobrycon heterorhabdus* (Ulrey 1894), popularly known as the Flag Tetra, is a widely distributed Amazonian species inhabiting streams throughout the biome (Prudente et al. 2018; Santos et al. 2019). This omnivorous, nektonic fish primarily feeds on insects (Brejão et al. 2013; Benone et al. 2020) and is suitable to diverse environmental disturbances, making it a potential model organism for ecotoxicological studies. Recent genomic and cytogenetic evidence reinforces its potential as a biomonitor for Amazonian ecosystems (Cardoso et al. 2025), and its mitochondrial genome has been sequenced (Montag et al. 2023), as well as its use as a model organism for assessing contaminants in the Amazon region (Nascimento et al. 2025). Furthermore, it is a fish of economic interest, as it is a popular freshwater ornamental fish (Novák et al. 2020).

Thus, this study aims to expand the use of *H. heterorhabdus* as a biological model, by developing and validating an integrated electrophysiological protocol capable of detecting sublethal physiological disorders in this species. Here, we assessed cardiac and muscular activity under normal conditions, during short-term exposure to a model xenobiotic (menthol), and during subsequent recovery to provide a replicable methodological framework with direct relevance for ecotoxicological studies in Amazonian freshwater ecosystems.

## Materials and methods

### Experimental animals and acclimation

Adult male specimens of *Hyphessobrycon heterorhabdus* (*N* = 63; 1.6 ± 0.15 g) were collected from a stream in the Gunma Ecological Park, located in the municipality of Santa Bárbara, Pará, Brazil, under the SISBio collection license 63470-2. The fish were collected using a 55 cm hand net with a mesh size of 2 mm between opposite knots, with a sampling duration of 15 min. After capture, fish were transported in aerated containers filled with stream water to Experimental Animal Facility of the Laboratory of Pharmacology and Toxicology of Natural Products at Federal University of Pará and maintained in glass aquaria (37 L) under controlled temperature and a 12 h light:12 h dark photoperiod. During the acclimatization process in the laboratory, partial water changes were performed in the aquariums over the course of several days to completely replace the stream water with water produced in the laboratory.

Fish were fed twice daily with a commercial feed for tropical fish containing 32% crude protein, offered to apparent satiation. During routine maintenance, uneaten food and feces were siphoned, and approximately 20% of the tank water was replaced with water from the same supply source. Fish were acclimated for three days prior to experimentation. During this period, water quality variables were monitored and maintained at the target values: water temperature 26 ± 0.97 °C; pH: 7 ± 0.71; dissolved oxygen > 5.0 mg L⁻¹; ammonia (NH_3_^+^) 0.1 ± 0.02 mg L⁻¹; and total hardness: 70 mg L⁻¹ CaCO_3_. The total number of individuals collected (*N* = 63) were divided for the behavioral experiment (*N* = 27) and electrophysiological analysis (*N* = 36).

### Drug acquisition and ethics agreement

Menthol (BIODINÂMICA^®^, Brazil) in crystalline form is a hydrophobic substance. A stock solution was prepared by diluting it in ethanol (96%) to a concentration of 100 mg mL^− 1^ (10%). Aliquots of this stock were diluted in water to obtain the desired anesthetic concentrations. The stock solution was stored in amber glass bottles at 4 °C until use.

All experimental procedures were conducted in accordance with institutional ethical guidelines and were approved by the Ethics and Animal Use Committee of the Universidade Federal do Pará (Protocol No. 7369290224).

### Experimental conditions

24 h before beginning of the experiments, the animals were acclimated individually in 1 L glass beakers containing only water, and feeding was stopped. During the exposure, each animal was kept in the container with the respective experimental solution. The experimental media used in all groups were prepared with reverse osmosis water reconstituted with salts (CaSO4 0.03 g/L; KCl 0.002 g/L; NaHCO3 0.0048 g/L; MgSO4 0.061 g/L; pH = 7), dissolved oxygen levels above 4 mg L⁻¹, and room temperature at 25 °C according to the recommendations of the standards for acute toxicity tests with fish of the Brazilian Association of Technical Standards NBR 15,088 (ABNT, 2022).

No deaths were recorded during exposure, and after completion of the experiment, the animals were anesthetized and euthanized with a lethal dose of lidocaine hydrochloride (350 mg/L) (Collymore et al. 2014).

### Behavioral experiment

**Fig. 1:**
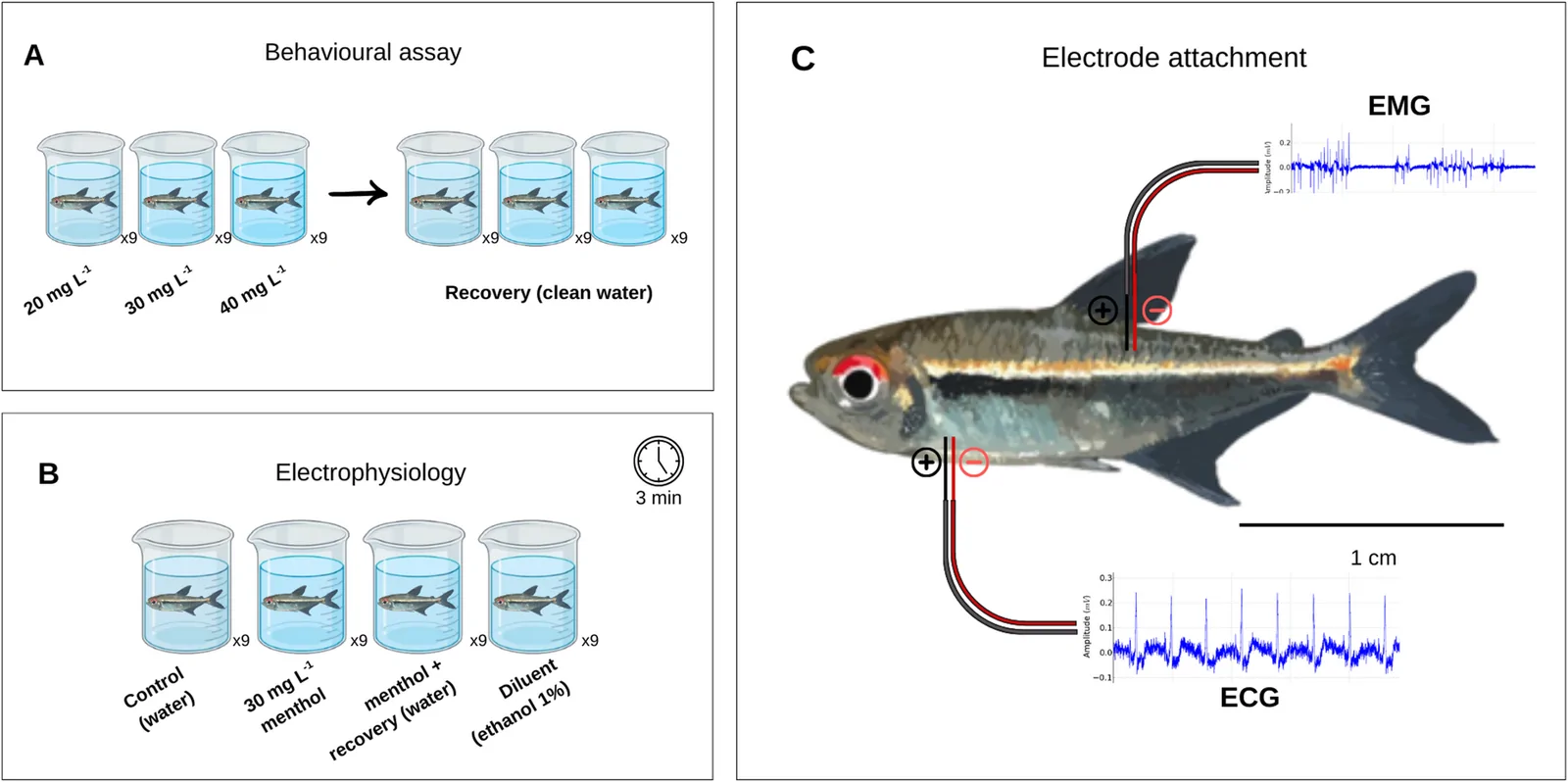
Experimental design of **A** behavioral and **B **electrophysiological assays, and **C** methodological scheme of electrode attachment in *Hyphessobrycon heterorhabdus*

Behavioral analysis consisted of quantifying the latency period for loss of posture reflex, characterized by loss of balance and erratic swimming, lateral recumbency maintained for more than 10 s, and loss of reaction to tail pinch stimulation (Barbas et al. 2017). Fish were divided into three groups with different concentrations of menthol: 20 mg L^−1^, 30 mg L^−1^, and 40 mg L^−1^, with nine animals per group (*N* = 27) (Fig. 1A). Subsequently, the animals were removed from the menthol and then placed in clean water to evaluate the recovery period of the posture reflex, characterized by maintaining the posture for a period longer than 10 s, and reaction to tail pinch stimulation. The observation of the experiment lasted until all the fish lost their posture. Since water and ethanol (1%) do not induce anesthesia—and thus do not result in the loss of the postural reflex—we considered only anesthetic induction using different concentrations of menthol.

### Electrophysiological experiment and markers

Fish (*N* = 36) were randomly assigned to the following treatment groups: (a) Control group, which was immersed in clean water; (b) Vehicle control group, with fish immersed in water with ethanol (1%), the solvent used for diluting menthol; (c) Group subjected to an immersion bath in menthol at a concentration of 30 mg L^− 1^; (d) Group immersed in menthol 30 mg L^− 1^ that was immediately allowed to recover in clean water (without ethanol), named recovery group (the animals in this group are not the same as those in the group exposed only to menthol) (Fig. 1B). The concentration of 30 mg L^− 1^ was chosen following the results of the behavioral assay performed previously (see results). Electromyographic (EMG) and electrocardiographic (ECG) recordings were conducted in parallel for each group, lasting three minutes each. A total of nine fish were used per treatment, resulting in 36 fish overall. For EMG and EGC recordings, the fish of all groups were placed on Petri dishes containing wet cotton wool and cold water for positioning the electrodes as described in the following sections. Recording was started 10 s after the fish were immersed in their respective experimental solutions to minimize any noise caused by handling the animals. During the experiment, the water temperature was kept constant, similar to the fish’s acclimation period.

#### Electromyographic recording (EMG)

Electrodes were constructed using two identical stainless-steel rods. One rod served as a reference, while the other was used for data recording. The rods measured 3.0 mm in length and had a diameter of 0.02 mm. They were insulated with Teflon (Microprobe SNC-MPI, Gaithersburg, MD 20879, USA) and coated with a thin layer of epoxy resin. The electrodes were attached to the dorsal muscle fibers, located 2.0 mm below the middle section of the dorsal fin (Fig. 1C). The distal parts of the electrodes were then connected to a high-impedance amplifier.

#### Electrocardiographic recording (ECG)

Electrodes were made of stainless steel, with a diameter of 200 micrometers and a length of 2.0 mm, in a non-conjugated form. The reference electrode was positioned based on the direction of the cardiac vector; it was carefully placed 2.0 mm ventrally via the right operculum in the craniocaudal direction (Fig. 1C). The recording electrode was placed in the same position but on the left side. After placement, the electrodes were connected to a high-impedance amplifier. From the recordings, the following parameters were analyzed: heart rate (bpm), amplitude (mV), QRS complex duration (s), PQ interval (s), RR interval (s), and QT interval (s).

#### Signal acquisition and processing

Electrodes were connected to a digital data acquisition system through a high-input impedance differential amplifier (Grass Technologies, Model P511). This amplifier was configured with filtering settings of 0.3 Hz and 300 Hz, and it provided 2000x amplification. The signal was monitored using an oscilloscope (ProteK, Model 6510). The recordings were continuously digitized at a rate of 1 kHz on a computer equipped with a data acquisition card (National Instruments, Austin, TX). The data was stored on a hard drive and subsequently processed using specialized software (LabVIEW Express).

Signals analyses were conducted using a tool developed in Python version 2.7. For mathematical processing, the Numpy and Scipy libraries were employed, while the Matplotlib library was utilized to generate graphs. The graphical interface was created using the PyQt4 library (Cantanhêde et al. 2020). The amplitude graphs illustrate the potential differences between reference and recording electrodes. The recording signals were sampled at a rate of 1,000 samples per second.

### Statistics analysis

The assumptions of normality (Kolmogorov-Smirnov test) and homogeneity (Levene’s test) of variances were evaluated, and after accepting the assumptions (*p* > 0.05), differences among groups were analyzed using one-way ANOVA, followed by Tukey’s post hoc test. A p value < 0.01 was considered statistically significant in all cases.

## Results

### Behavioral responses

**Fig. 2:**
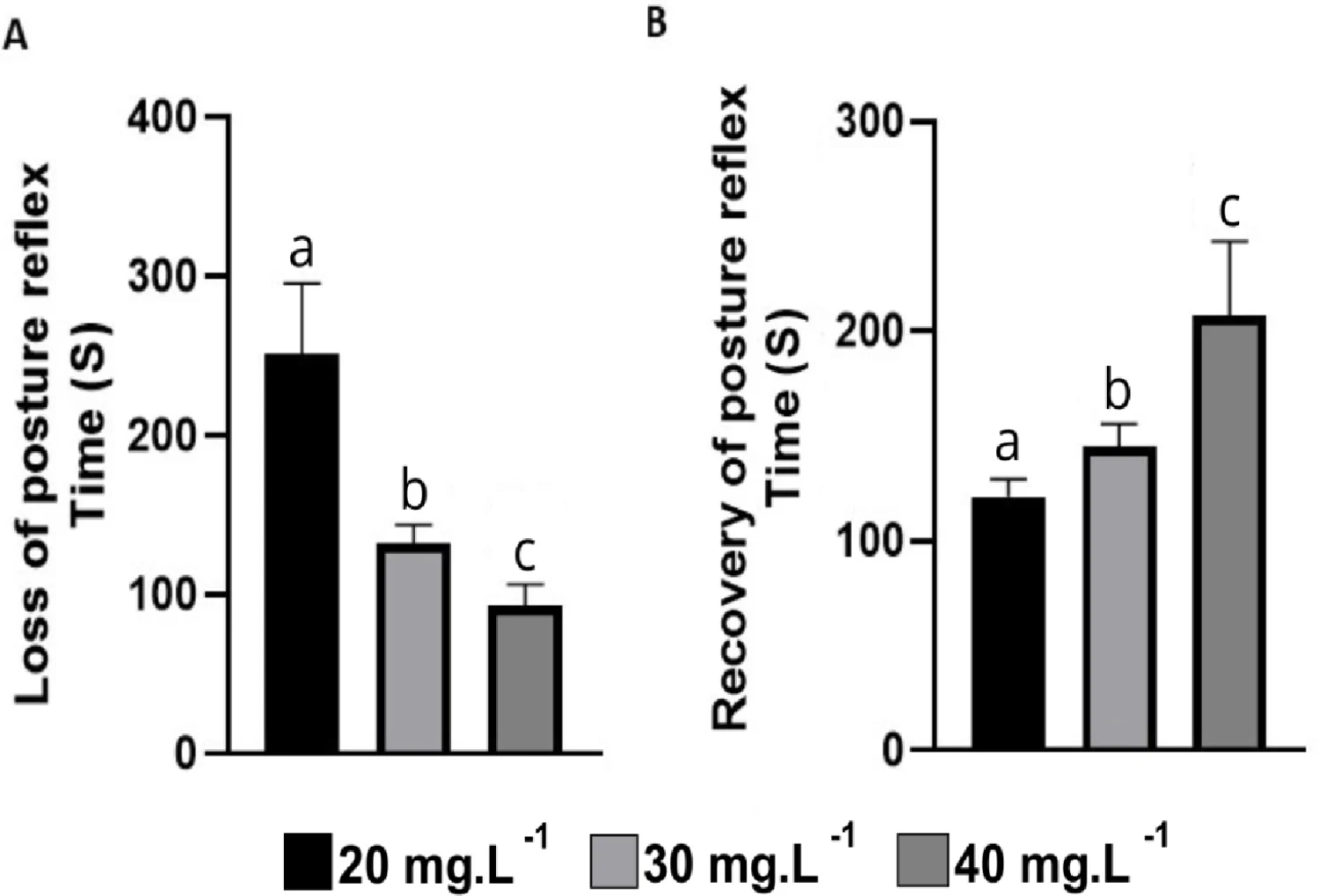
Behavioral analysis of *Hyphessobrycon heterorhabdus* exposed to different concentrations of menthol. **A** Analysis of mean latencies (seconds) for loss of posture reflex during immersion baths in different concentrations of menthol. **B** Time for recovery of posture reflex after contact with different concentrations of menthol in immersion baths. Different letters indicate significant differences. Note the difference in the scales of the graphs

Exposure to menthol induced a progressive loss of the righting reflex, with significant differences among all treatments (F(2,24) = 77.45; *P* < 0.0001) (Fig. 2A). The animals showed loss of posture reflex at 250.30 ± 44.98 s (20 mg L^− 1^), 131.60 ± 11.82 s (30 mg L^− 1^), and 92.00 ± 14.28 s (40 mg L^− 1^). A similar concentration-dependent pattern was observed for the recovery of the posture reflex after 3 min of contact with menthol, where the animals showed recovery periods of 121.20 ± 8.19 s, 144.9 ± 10.73 s, and 207.20 ± 35.54 s for 20, 30, and 40 mg L^− 1^ of menthol, respectively (F(2,24) = 36.86; *P* < 0.0001) (Fig. 2B). The concentration of 30 mg L^− 1^ indicates an acceptable period of time for electrode attachment when performing the analysis of electrophysiological markers.

### Electromyography

**Fig. 3:**
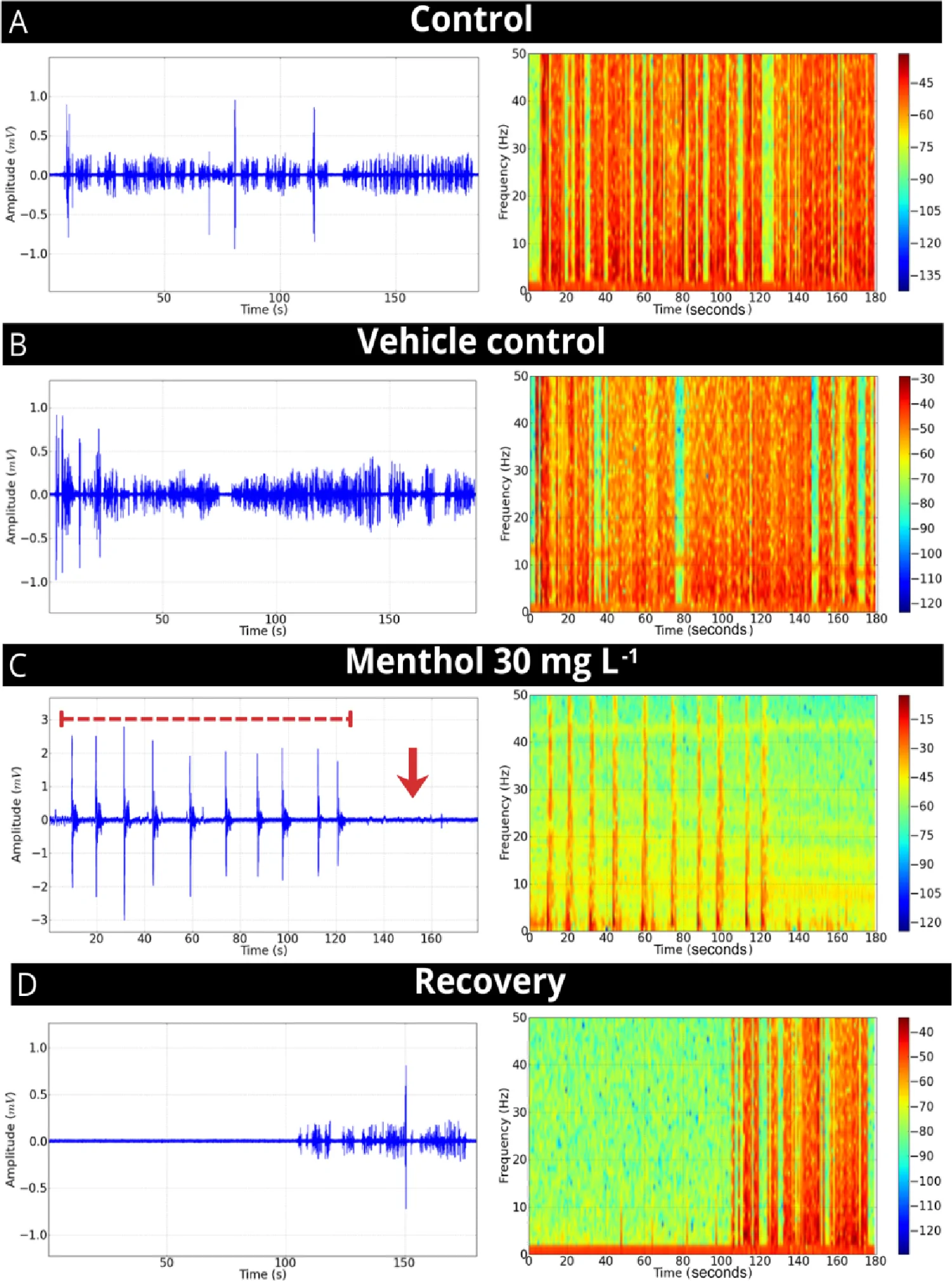
Electromyographic (EMG) tracings of the dorsal muscle of *Hyphessobrycon heterorhabdus* submitted to a 3-minute immersion bath with menthol at 30 mg L^− 1^. **A** Control recordings. **B** Fish exposed to vehicle (ethanol) in water. **C** Fish exposed to 30 mg L⁻¹ menthol. **D **Fish recovering after contact with menthol. EMG over 180 s (left), and frequency spectrograms (right). Muscle spasms of high magnitude during induction were highlighted with a red line, and the period of anesthetic induction was indicated by a red arrow

**Fig. 4:**
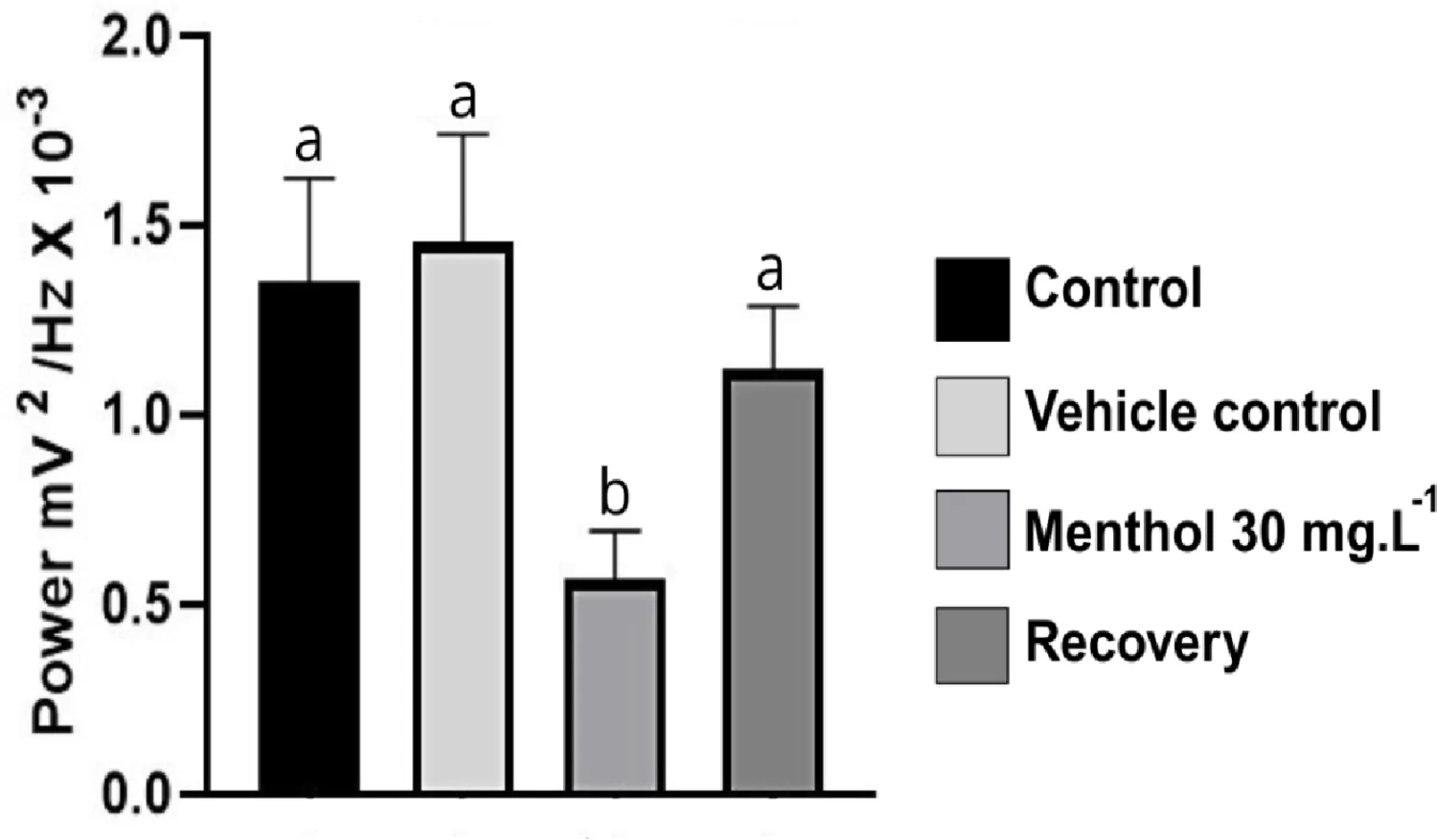
Electromyographic recordings (mean amplitude ± SE) (EMG) in the dorsal muscle of *Hyphessobrycon heterorhabdus* (*n* = 9). All recordings were performed for 180 s, including controls. Mean amplitudes are shown for the entire induction period. Different letters indicate significant differences. Note the difference in the scales of the graphs

Electromyographic recordings of the control fish showed high amplitude traces and concentrated energy intensity at frequencies exceeding 50 Hz, where frequency spectrograms show rapid contraction of the dorsal muscle of *H. heterorhabdus* (Fig. 3A) with an average power of 1.352 ± 0.2722 mV^2^ / Hz 10^− 3^. The EMG parameters of the vehicle-exposed group displayed a similar electrophysiological profile, showing no signs of muscle excitability or relaxation (Fig. 3B), with a mean power of 1.458 ± 0.2832 mV²/Hz 10⁻³ (Fig. 4).

Fish immersed in menthol initially exhibited high-amplitude muscle spasms and transient muscle spasms, indicated by a red arrow, before progressing to a stage of muscular relaxation (Fig. 3C). The increase in amplitude is shown in the spectrogram, where the energy distribution was initially elevated by the spasms and subsequently decreased, reflecting a significant reduction in the mean power (0.5698 ± 0.1246 mV^2^/Hz 10^− 3^) compared to the control group (Fig. 4).

During recovery, fish progressively regained muscle tonus approximately 100 s after transfer to clean water (Fig. 3D). Electromyographic activity and mean power (1.119 ± 0.1696 mV^2^/Hz 10^− 3^) returned to levels comparable to the control and vehicle groups (Fig. 4), indicating full reversibility of menthol-induced neuromuscular effects.

### Electrocardiography

**Fig. 5:**
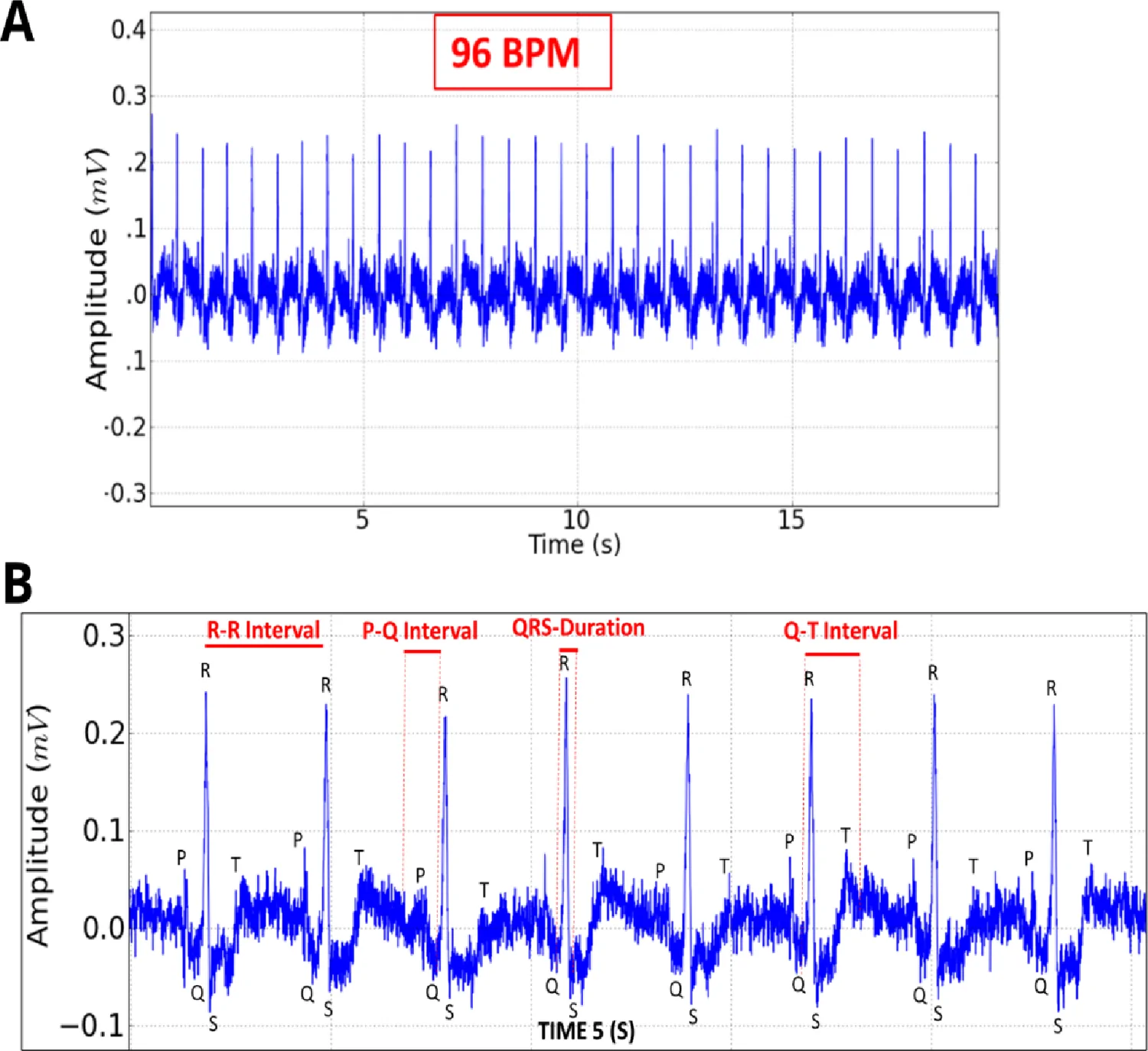
Normal electrocardiogram (ECG) of *Hyphessobrycon heterorhabdus* (*n* = 9) (**A**). Expanded electrocardiogram showing the data analyzed: P-Q interval, R-R interval (S), QRS duration (S), Q-T interval (S) (**B**)

Baseline cardiac parameter recordings from the *H. heterorhabdus* showed a well-defined sinus rhythm (Fig. 5A), with a mean heart rate of 101.6 ± 5.457 bpm. All waves (P, Q, R, S, T), intervals (PQ, RR, QT), and QRS complexes are visible (Fig. 5B), providing a robust reference for subsequent comparisons.

**Fig. 6:**
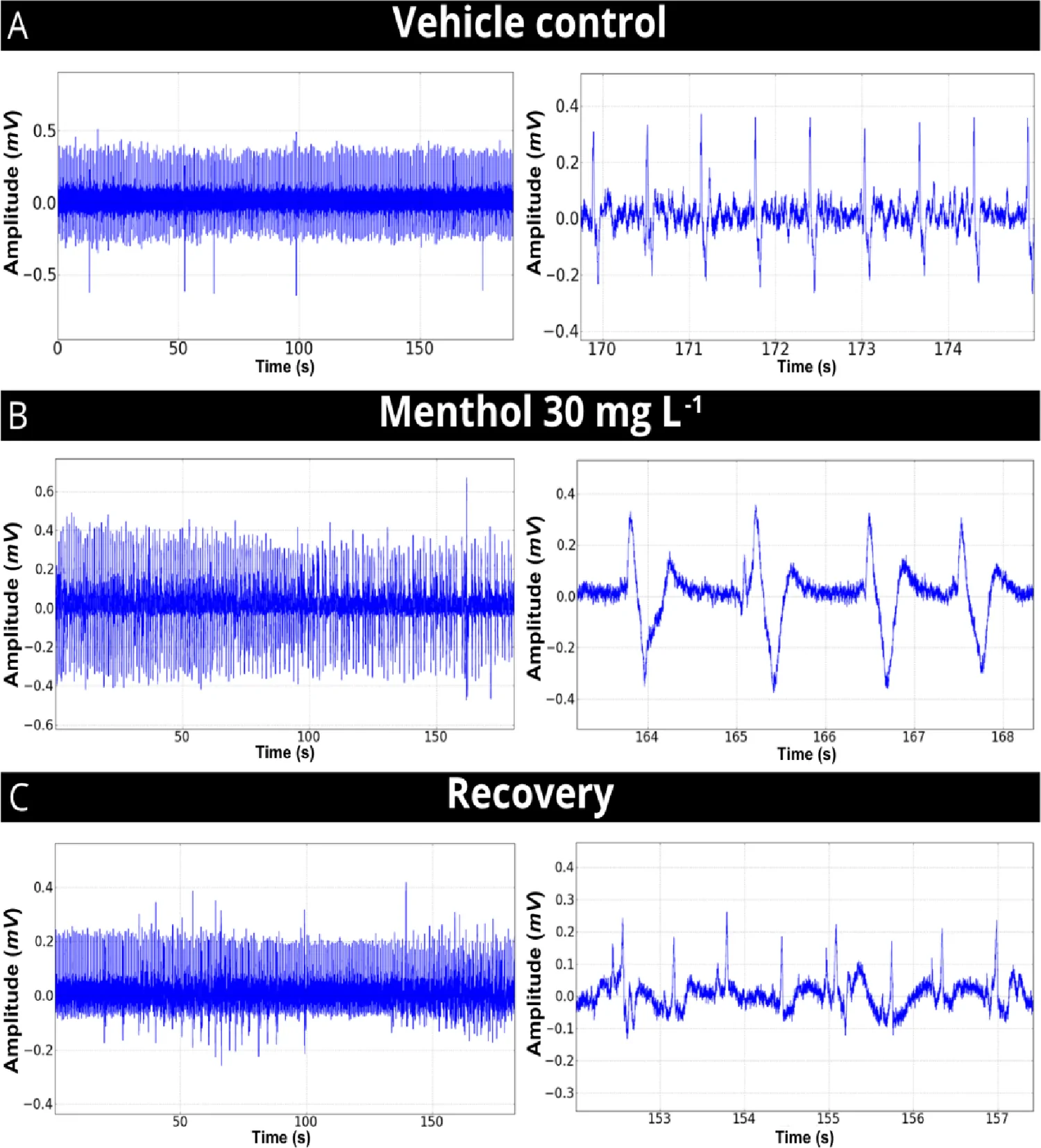
Electrocardiogram (ECG) recording of *Hyphessobrycon heterorhabdus* after exposure and recovery from menthol for 3 min (*n* = 9). **A** Recording of fish in vehicle control (ethanol 1%; left) and its amplification (right). **B** Recording of fish immersed in 30 mg L⁻¹ of menthol (left) and its amplification (right). **C **Recovery from the menthol immersion bath (left) and its amplification (right)

The ECG of *H. heterorhabdus* in the vehicle control group was similar to that of the control group (Fig. 6A). Short-term electrocardiographic changes were observed during the fish’s exposure to menthol (30 mg L⁻¹; Fig. 6B). According to the observed changes, the main variations in the trace occur in the second half of the recording (90–180 s), such as a decrease in heart rate and the appearance of irregularities in the recording (Fig. 6B). Upon transfer to clean water, the fish progressively returned to a normal sinus rhythm (Fig. 6C).

**Fig. 7:**
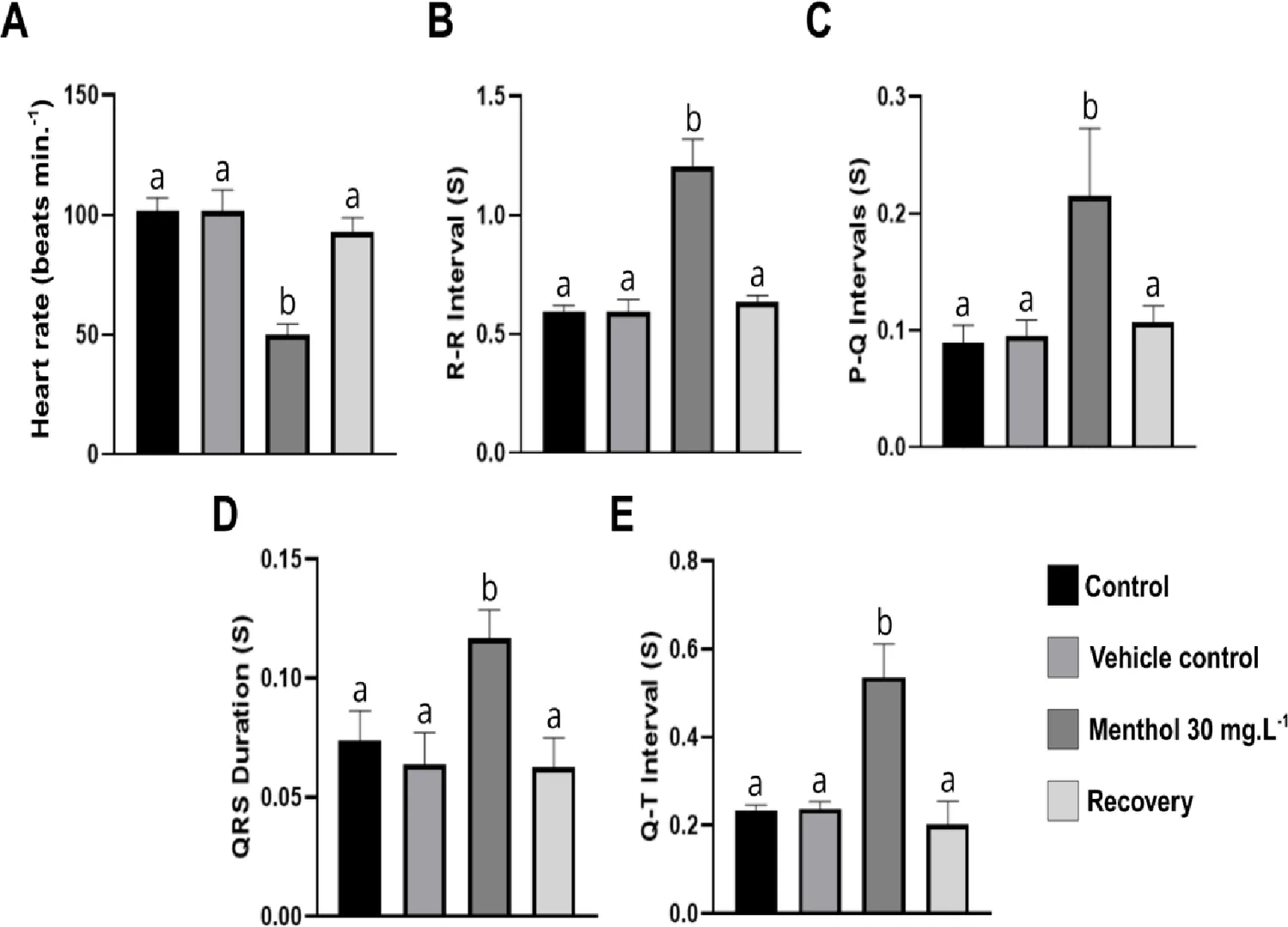
Electrocardiographic (ECG) parameters of *Hyphessobrycon heterorhabdus* following immersion in 30 mg L^-1^ menthol and recovery period. Data expressed as mean ± SE. Heart rate (bpm) (**A**). R-R intervals (**B**). P-Q intervals (**C**). QRS complex duration (**D**). Q-T interval duration (**E**). Different letters indicate significant differences. Note the difference in the scales of the graphs

Menthol exposure caused significant bradycardia. The group treated with 30 mg/L of menthol had a mean heart rate of 50.22 ± 4.295 bpm, which was significantly lower than that of the control group (101.6 ± 5.457 bpm), and the vehicle control group (101.4 ± 9.043 bpm), with no significant difference between the control groups. During recovery, heart rate increased to 92.89 ± 5.925 (bpm), a value statistically similar to those of the control groups and higher than that of the menthol-exposed group (F(2, 24) = 244.7; *P* < 0.0001) (Fig. 7A).

The RR interval in the control group was consistent, with a mean of 0.5955 ± 0.02441 s, showing no significant differences from the vehicle control (0.5950 ± 0.0550 s). When the animals were exposed to menthol, a noticeable separation between the R wave deflections was observed, leading to an increase in cardiac cycle duration, with a mean of 1.207 ± 0.1132 s. This value was significantly higher than that observed in the control groups. During the recovery phase, the mean RR interval was 0.6344 ± 0.02823 s, which was longer compared to the menthol-exposed group, although it was similar to the controls (F(2, 24) = 222.3; *P* < 0.0001) (Fig. 7B).

The PQ interval also increased significantly after exposure, reaching 0.2150 ± 0.05782 s, compared with 0.08956 ± 0.01514 s in the control group and 0.0956 ± 0.0133 s in the vehicle control group. Recovery values (0.1074 ± 0.01375 s) were similar to those of the control groups (F(2, 24) = 33.5; *P* < 0.0001) (Fig. 7C).

Fish that were anesthetized with menthol exhibited a significantly longer QRS complex duration (0.1168 ± 0.01191 s) compared to the control group (0.07379 ± 0.01247 s), the vehicle control group (0.06389 ± 0.01329 s), and the recovery group (0.06256 ± 0.01252 s) (F(2, 24) = 48.68; *P* < 0.0001) (Fig. 7D).

Finally, the QT interval increased substantially during exposure (0.5372 ± 0.07458 s), more than double the value observed in the control group (0.2347 ± 0.01191 s), and the vehicle control group (0.2383 ± 0.01564 s). After recovery, the QT interval duration (0.2013 ± 0.05367 s) was similar to that of the control groups (F(2,24) = 107.7; *P* < 0.0001) (Fig. 7E).

## Discussion

Anesthetic induction by menthol occurs through the blockade of voltage-dependent sodium channels in neurons and skeletal muscles (Haeseler et al. 2002; Fermini and Fossa 2003). Because of its well-characterized physiological actions, menthol represents a suitable reference compound for validating electrophysiological methodologies. In this study, menthol was used as a model substance to assess the application of electrophysiological biomarkers, especially the electromyogram and electrocardiogram, in the Amazonian fish *H. heterorhabdus*, thereby contributing to the establishment of this Amazonian species as a model organism for ecotoxicological and physiological studies.

Behavioral assays revealed a concentration-dependent reduction in the latency to loss of the righting reflex and a proportional increase in recovery time, demonstrating the effectiveness of menthol as an anesthetic agent for this species. The use of this reflex is one of the most used endpoints in behavioral studies involving anesthetics (Von Krogh et al. 2021), yet such evaluations, particularly in small native Amazonian species, remain scarce. Instead, research typically focuses on commercially important fish, aiming to identify concentration levels suitable for managing aquaculture stocks (Fujimoto et al. 2018; Garcia et al. 2024). In Brazil’s aquaculture systems, the Tambaqui *C. macropomum* is one of the most extensively studied species, with investigations into its behavior and electrophysiological responses following exposure to substances such as lidocaine, *Nepeta cataria* oil, and *Curcuma longa* oil (Vieira et al. 2023; Dos Santos et al. 2024; Cerqueira et al. 2025). The present results, therefore, expand the available data for a small-bodied Amazonian species with potential biomonitoring utility.

EMG analyses further supported the behavioral findings. Control fish displayed high amplitude and rapid muscle contractions, typical of active swimming (Cantanhêde et al. 2020). In their natural environment, *H. heterorhabdus* inhabits backwater areas near the banks of streams and moves through the water column in search of food (Brejão et al. 2013; Oliveira et al. 2023). During menthol exposure, fish exhibited transient high-amplitude muscle spasms, followed by decreased muscle activity, a pattern similar to that described in *C. macropomum* after exposure to citronella essential oil (Barbas et al. 2017) and *Piper divaricatum* (Vilhena et al. 2019). The subsequent restoration of EMG amplitude and power after transfer to clean water indicates a reversible muscular depression consistent with anesthetic-induced relaxation.

ECG recordings provided complementary evidence of reversible cardiac depression. There was clear identification of the P wave, representing atrial excitation; the QRS complex, indicating ventricular depolarization and potential change in cardiomyocytes; the T wave, representing ventricular repolarization; and the QT intervals, representing the total time of ventricular depolarization and repolarization; and R-R, representing the complete duration of the cardiac cycle (Reis et al. 2025). These patterns confirm the feasibility of ECG acquisition in *H. heterorhabdus*. Exposure to menthol induced bradycardia accompanied by prolonged RR, PQ, QT intervals and QRS duration—patterns indicative of slowed conduction, delayed depolarization, and extended repolarization (Reis et al. 2025).

The reduction in heart rate here is consistent with findings in other fish exposed to natural or synthetic anesthetics, which generally reduce cardiac output and metabolic demand (Cantanhêde et al. 2020). As expected, cardiac parameters returned to values similar to those of the control group after recovery, demonstrating that menthol’s short-term effects on cardiac physiology are reversible. Analogous cardiac alterations have been reported in *C. macropomum*, where menthol induced reversible changes in a concentration-dependent manner (ranging from 40 to 80 mg L⁻¹) in heart rate, QT, and RR intervals duration (Cantanhêde et al. 2021).

Since the mechanisms of action of anesthetic substances differ from those of numerous other potentially toxic substances, electrophysiological analyses should be performed with caution, and it is recommended that they be combined with other analyses at different levels of biological organization. Beyond detecting anesthetic-like responses, ECG endpoints are increasingly recognized as sensitive biomarkers of cardiotoxicity (Cantanhêde et al. 2020). Studies in Amazonian and non-Amazonian species have shown that metals such as manganese (Nascimento et al. 2024), methylmercury (Da Paz et al. 2025), aluminum (Cantanhêde et al. 2022), thallium (Song et al. 2018), and cadmium (Xing et al. 2017) can alter cardiac intervals and waveform morphology. Pharmaceutical compounds have been shown to have effects on the ECG in fish, such as increased QT interval after oral administration of antiallergics and antipsychotics (Millan et al. 2006), and exposure to antiarrhythmics such as Amiodarone (Yu et al. 2010) and Verapamil (Dhillon et al. 2013).

In addition, it is important to note that our study presents some limitations that should be considered. The sample size (*n* = 9) may be considered relatively small due to the inter-individual variability; however, the consistent EMG and ECG patterns observed support the reliability of the results. Future studies should expand this approach by incorporating complementary biomarkers to assess the reversibility and safe use of menthol in *H. heterorhabdus*, such as physiological stress indicators (cortisol), oxidative damage, and histopathological analyses. Nevertheless, the short duration of exposure observed in our study does not reveal any potential adverse effects resulting from subchronic exposure. This integrative approach, and a longer exposure time, would provide a more comprehensive assessment of ecotoxicological responses in future research.

## Conclusion

Menthol promoted transient and fully reversible electrophysiological alterations in *Hyphessobrycon heterorhabdus*, characterized by bradycardia, prolongation of ECG intervals (RR, PQ, QT) and widening of the QRS complex, as well as modulation of skeletal muscle activity. These effects were accompanied by muscle spasms during induction and a subsequent reduction in contraction power, but all parameters returned to baseline after removal of the menthol, here as a model xenobiotic, confirming the functional reversibility of the responses. The integrated analysis of electrocardiographic and electromyographic recordings proved to be a sensitive and robust tool for detecting acute physiological responses in small Neotropical fish, contributing to the advancement of experimental protocols involving stress assessment and early detection of xenobiotic toxicity. Our results contribute to broadening the understanding of acute physiological mechanisms in *H. heterorhabdus*, replicable in future studies to evaluate the effects of exposure to environmental stressors within the Amazonian context.

## Data Availability

Data will be made available upon request.
